# A neuroimaging study of brain activity alterations in treatment-resistant depression after a dual target accelerated transcranial magnetic stimulation

**DOI:** 10.3389/fpsyt.2023.1321660

**Published:** 2024-01-15

**Authors:** Jiaoying Liu, Yanping Shu, Gang Wu, Lingyan Hu, Hailun Cui

**Affiliations:** ^1^Department of Clinical Medicine, Zunyi Medical University, Zunyi, China; ^2^Department of Psychiatry, The Second People's Hospital of Guizhou Province, Guiyang, China; ^3^Department of Psychiatry, University of Cambridge, Cambridge, United Kingdom

**Keywords:** treatment-resistant depression, transcranial magnetic stimulation, resting-state functional magnetic resonance imaging, amplitude of low-frequency fluctuations, fractional amplitude of low-frequency fluctuations, functional connectivity

## Abstract

In this study, we designed a new transcranial magnetic stimulation (TMS) protocol using a dual-target accelerated transcranial magnetic stimulation (aTMS) for patients with treatment resistant depression (TRD). There are 58 TRD patients were recruited from the Second People’s Hospital of Guizhou Province, who were, respectively, received dual-target (real continuous theta burst stimulation (cTBS) at right orbitofrontal cortex (OFC) and real repetitive transcranial magnetic stimulation (rTMS) at left dorsolateral prefrontal cortex (DLPFC)), single- target (sham cTBS at right OFC and real rTMS at left DLPFC), and sham stimulation (sham cTBS at right OFC and sham rTMS at left DLPFC). Resting-state functional magnetic resonance imaging (rs-fMRI) was acquired before and after aTMS treatment to compare characteristics of brain activities by use of amplitude of low-frequency fluctuations (ALFF), fractional low-frequency fluctuations (fALFF) and functional connectivity (FC). At the same time, Hamilton Depression Scale-24 (HAMD_24_) were conducted to assess the effect. HAMD_24_ scores reduced significantly in dual group comparing to the single and sham group. Dual-target stimulation decreased not only the ALFF values of right fusiform gyrus (FG) and fALFF values of the left superior temporal gyrus (STG), but also the FC between the right FG and the bilateral middle frontal gyrus (MFG), left triangular part of inferior frontal gyrus (IFG). Higher fALFF value in left STG at baseline may predict better reaction for bilateral arTMS. Dual-targe stimulation can significantly change resting-state brain activities and help to improve depressive symptoms.

## 1 Introduction

Depression, manifested as marked and persistent low mood that is not commensurate with the environment, is a serious global public health problem. In recent years, with the increasing pressures of modern life and continuous improvement in disease diagnosis skill, it is found that the population of patients suffering from depression is consistently expanding worldwide. According to a World Health Organization (WHO) report (1), there are approximately 350 million patients with depression of different ages currently. This highly prevalent disorder also has high rates of disability, recurrence, mortality, and heritability (2, 3). As a result, individuals, family, and society need to face the huge economic and emotional burden imposed by the recurrence and sequelae of depression. Given the damage to the public, improving treatments and extending the long-term prognosis of depressive patients are urgently needed.

At present, the main treatment methods for depression include medication psychotherapy and physical therapy. However, approximately one-third to two-thirds of patients still do not respond to medication, which is known as treatment-resistant depression (TRD) patients (4–7). TRD is typically defined as low efficacy of two or more antidepressants with different chemical structures at adequate dosages and courses (8–10). Therefore, in recent years, researchers have begun to explore another method, especially physical one, to improve capacity for treating TRD. Some of these studies reported that nonpharmacological treatments have a durable effect than medication (11–13). Among the nonpharmacological therapies, transcranial magnetic stimulation (TMS) is a safe, non-invasive, and painless way that was approved by the U.S. Food and Drug Administration (FDA) in 2008 for the treatment of depression (14). However, traditional repetitive transcranial magnetic stimulation (rTMS) treatment usually takes several weeks and shows limited efficacy. To further improve the treatment efficiency and exclude the influence of other factors, such as drugs and psychotherapy, some researchers have begun to pay their attention to accelerated repetitive transcranial magnetic stimulation (arTMS). Because it could compress the treatment cycle from weeks into a few days while maintaining safety and effectiveness, arTMS not only greatly reduces the suffering of patients but also decreases the time required for treatment and improves efficiency (15).

The brain of human is a complicated structure, because different brain regions perform their own functions and coordinate to complete complex functions, such as perception, processing, and action execution etc. Quite a few numbers of studies have been committing to detect some brain areas as the stimulation targets which are related to the effect of TMS and to improve the efficacy of TMS for treating TRD by using different stimulation parameters. At present, the FDA has approved the TMS targeted at the left dorsolateral prefrontal cortex (DLPFC), which is a key area in depressive symptoms and shown to be hypoactive in major depressive disorder, for the treatment of depression (16–18). The efficacy of this traditional protocol is limited even though it takes several weeks. Considering that the effect of current treatment protocols is still limited, it is needed to explore more possible programs. Some new studies showed that bilateral rTMS treatment was more effective for treating depressive symptoms than unilateral, sham stimulation or medicine only (19, 20). We noticed that the lateral orbitofrontal cortex (lOFC), related to non-reward system, is implicated in the rumination of sad events and memories in depression patients this indicated that the lOFC is a crucial target for the improvement of depression (21–25). Thus, this study researched the effect of a new stimulation protocol targeted at the left DLPFC and right lOFC and the changed of functional magnetic resonance imaging (fMRI) before and after the treatment.

Resting-state functional magnetic resonance imaging (rs-fMRI) has been used to explore the spontaneous activity in brains and neurobiological mechanisms in depressed patients by diverse analysis methods which have been mainly divided into the functional segregation and integration (26–28). The former includes amplitude of low-frequency fluctuations (ALFF), fractional amplitude of low-frequency fluctuations (fALFF), etc. In 2007, Zang et al. suggested that the ALFF, which assessed oscillation by measuring the blood-oxygen-level dependent (BOLD) signals in the low-frequency range (0.01 ~ 0.08 Hz) based on the voxel level, reflected spontaneous neural activity in specific brain areas (29). Compared with other methods of analysis, fractional amplitude of low-frequency fluctuations (fALFF) is a more useful way for measuring the spontaneous activity of the resting brain with fewer physiological noise and nonspecific signals (30). The later embraces graph theory, seed-based analysis, independent component analysis (ICA) and so on (26). Graph theory describe the relationship between nodes and edges depend on node degree, centrality, average path length, etc. (31–34), where nodes can be the electrodes and channels of electroencephalogram and magnetoencephalography or the common region of interest (ROI) defined on structural and functional template, while the edge refers to connections between the nodes (35). Seed-based analysis mainly focuses on the correlation between one ROI to another one refers to the synchronous activity between different brain regions and indicates whether these two brain regions are related in terms of function (26, 29). ICA helps to extract different networks and analysis simultaneous voxel to voxel interactions among networks (26, 28). In this study, our team will analyze the differences in brain activity of depressive patients before and after receiving different arTMS from the both perspectives of separation and integration.

We conducted arTMS treatment for the TRD patients aimed at left DLPFC and right lOFC, a brain area related to the reward mechanisms, and collected brain functional images of subjects before and after treatment using rs-fMRI. Then, the neural activity was evaluated by ALFF, fALFF, and FC value before and after arTMS.

## 2 Methods

### 2.1 Participants and groups

This study was approved by the Ethics Committee of the Second People’s Hospital of Guizhou Province. From August 2021 to July 2022, 60 patients with TRD were recruited from this hospital. Subjects or their legal guardians agreed to participate in this study and signed the informed consent form. This trial was prospectively registered in the China Clinical Trial Registry (Registration number: ChiCTR2100049002).

The inclusion criteria were as follows: (1) met criteria of the Diagnostic and Statistical Manual of Mental Disorders, fifth edition (DSM-V) diagnostic for MDD; (2) were right-handed; (3) were 18 to 60 years old (regardless of sex); (4) had an HAMD24 score ≥ 21; (5) previously received a full course of two or more antidepressant drugs at a sufficient dosage but achieved little or no treatment response; and (6) signed informed consent form.

The exclusion criteria were as follows: (1) with psychotic symptoms or any other mental disorders; (2) with symptoms caused by organic diseases or medications; (3) with severe organic disease; or (4) with contraindications for TMS or MRI, such as a history of epilepsy, pregnancy within 3 months, an artificial heart valve or a pacemaker.

### 2.2 Intervention

In this study, we adopted the following arTMS treatment protocol to reduce the traditional 4–5 weeks period of stimulation to 5 days by delivering multiple stimulations per day. The specific procedure was as follows: first, cTBS at 5 Hz was applied to the right OFC for a total of 48 s, which included 600 pulses and a resting motor threshold (RMT) of 100% ± 10%. Then, high-frequency repetitive transcranial magnetic stimulation (HF-rTMS) at 20 Hz was applied to the left DLPFC for 90 s, which included 1,800 pulses and an RMT of 100% ± 10%. The above stimulations were performed 4 times a day, with 50-min intervals between each series, for 5 consecutive days. Thus, the right OFC target received a total of 12,000 pulses, and the left DLPFC target received a total of 36,000 pulses during the full course.

Patients in these three groups received different arTMS stimulations with the same parameters described above. In the dual group, real stimulation was applied to both the right OFC and left DLPFC. However, in the single group, the subjects received sham stimulation treatment with a fake figure-eight coil that mimicked the real one at right OFC and received real stimulation at the left DLPFC. In the sham group, the subjects received sham stimulation treatment with a fake figure-eight coil at both the right OFC and left DLPFC.

### 2.3 Assessment indicators

All subjects completed the Hamilton Depression Scale-24 (HAMD24), which is widely used in clinical diagnosis because of its good reliability and validity, before and after arTMS intervention, 1 week after intervention, and 4 weeks after intervention to assess the depressive symptoms. Before and after arTMS, resting-state fMRI was used to observe spontaneous brain activity in fALFF and fALFF in different brain regions. Then, regions with significant differences after arTMS were selected as regions of interest (ROIs), and with this as the center, ROI of *r* = 6 mm was used as the seed point, and the voxel-wise functional connectivity of TRD depression patients was calculated.

### 2.4 Image acquisition

The acquisition of rs-fMRI image data in this study was completed by professional technicians with intermediate or higher professional titles in the imaging department in an examination room. The data were collected by a high-field magnetic resonance scanner from GE (manufacturer’s model: SIGNA HDe) (Coil: General Electric, Madison, WI, USA).

During the scan, all patients were asked to remain calm and awake; keep their eyes closed; and refrain from moving their head. The collection of rs-fMRI data in this study utilized the following two specific scanning sequences: (1) 3D-T1-weighted whole-brain structure imaging with a fast spoiled gradient echo (FSPGR) sequence (slices = 116, slice thickness = 1.2 mm, repetition time (TR) = 12.536 ms, echo time (TE) = 5.432 ms, inversion time = 350, flip angle (FA) = 20°, and matrix = 256 × 256). (2) resting-state fMRI using a gradient echo and echo planer imaging (GRE-EPI) sequence (slices = 28, slice thickness = 3.5 mm, TR = 2,500 ms, TE = 40 ms, time points = 300, FA = 90°, and matrix = 80 × 80).

### 2.5 Rs-fMRI data processing

Related data were analyzed by Data Processing and Analysis for Brain Imaging (DPABI) software (36), which was based on the MATLAB_2013b environment. The following 12 steps were used: (1) conversion from DICOM to NIFTI, (2) removal of the first ten time points to reduce inaccuracy resulting from head movement or other factors at the beginning of scans, (3) slice timing correction to preventing interference between adjacent slices by adopting interval scanning, (4) realignment (checking and correcting the head motion), (5) nuisance regression (removing another covariate), (6) transformation to Montreal Neurological Institute (MNI) space, (7) detrending (removing the noise of the machine), (8) smoothing (reducing the effects of spatial noise and reducing differences in brain structure between subjects), (9) calculation of ALFF (0.01–0.08 Hz) and fALFF values to reflect the spontaneous resting-state activity of each different brain region (ALFF and fALFF maps were standardized by z score-transformation into zALFF and zfALFF maps), (10) quality control (evaluating the quality of images and excluding the subjects whose images did not meet the quality requirements), and (11) calculation of FC (brain regions with the cluster voxels size >40 in the corrected ALFF and fALFF results were selected as ROIs for further analysis of the FC between the ROIs and whole brain).

### 2.6 Statistical analysis

SPSS 29.0 software was used to perform the chi-square test to assess the influence of sex on the three groups, and one-way analysis of variance (ANOVA) was performed to explore the influence of age, education level and HAMD24 scores of the three groups. A *p* value less than or equal to 0.05 was considered statistically significant. We extracted the time courses of brain regions with abnormal ALFF and fALFF values and then conducted correlation analysis in SPSS software to calculate the Pearson correlation coefficient between the difference in HAMD24 scores (∆HAMD24) with ALFF or fALFF.

The DPABI software (36) was used to perform statistical analysis of images with the following steps: (1) paired *t* test: To calculate ALFF and fALFF values and to identify ROIs that significantly differed at pre-TMS and post-TMS in the three groups, we applied the paired t test in DPABI software (36). (2) Multiple comparisons: gaussian random field (GRF) correction with a voxel *p* value of 0.002 and cluster p value of 0.1 was used to determine two-tailed significant differences to reduce the probability of type I error. (3) The brain regions with cluster voxels size greater than 40 were selected.

### 2.7 Visualization

The REST V1.8 software^1^ (37) and BrainNet Viewer^2^ (38) were used to visualize the results of brain activity and networks.

## 3 Results

A total of 60 participants were enrolled, but 2 subjects did not complete the full treatment course. Finally, 58 patients (46 females and 12 males, aged 18–56 years) had received 5-day treatment and 4-week follow-up. All included subjects, who were marched for gender, age, and education level, were randomly divided into three groups as follows by computer randomization sequences: the dual target group (dual group) (19 subjects, aged 27.58 ± 9.605 years), single target group (single group) (19 subjects, aged 26.32 ± 8.845 years) and sham stimulus group (sham group) (20 subjects, aged 28.70 ± 10.887 years) (Table 1).

**Table 1:** Comparison of HAMD24 scores among dual, single and sham groups.

Variables	dual (*n* = 19) Mean ± SD	single (*n* = 19) Mean ± SD	sham (*n* = 20) Mean ± SD	*p* value
Age (years)	27.58 ± 9.605	26.32 ± 8.845	28.70 ± 10.887	0.752^a^
Gender (male/female)	4/15	3/16	5/15	0.776^b^
Education (years)	4.80 ± 6.815	4.66 ± 5.800	2.38 ± 1.804	0.272 ^a^
HAMD_24_ (pre)	49.16 ± 10.150	45.16 ± 10.156	50.80 ± 9.134	0.194^a^
HAMD_24_ (Post)	29.11 ± 12.405	30.42 ± 10.543	43.50 ± 8.618	<0.001^a,^
HAMD_24_ (1 week)	23.26 ± 11.685	26.68 ± 9.563	37.15 ± 10.266	<0.001^a,^
HAMD_24_ (4 weeks)	22.21 ± 12.196	22.58 ± 9.353	34.80 ± 12.685	0.001^a^
reduction (before-after)^c^	41.67% ± 0.239	29.70% ± 0.368	13.43% ± 0.151	0.006^a^
reduction (before-1 week after)^d^	53.24% ± 0.231	37.72% ± 0.350	27.00% ± 0.162	0.010^a^
reduction (before-4 weeks after)^e^	55.47% ± 0.219	47.17% ± 0.333	32.49% ± 0.211	0.025^a^

SD, Standard Deviation; HAMD24, Hamilton Depression Scales-24.

^a^The p value for one-way ANOVA.

^b^The p value for Chi-square test.

^c^Reduction rate of HAMD24 scores for before and after treatment = ((pre-post)/pre).

^d^Reduction rate of HAMD24 scores for before and 1 week after treatment = ((pre-1 week)/pre).

^e^Reduction rate of HAMD24 scores for before and 4 weeks after treatment = ((pre-4 weeks)/pre).

### 3.1 HAMD24 analysis

**Figure 1:**
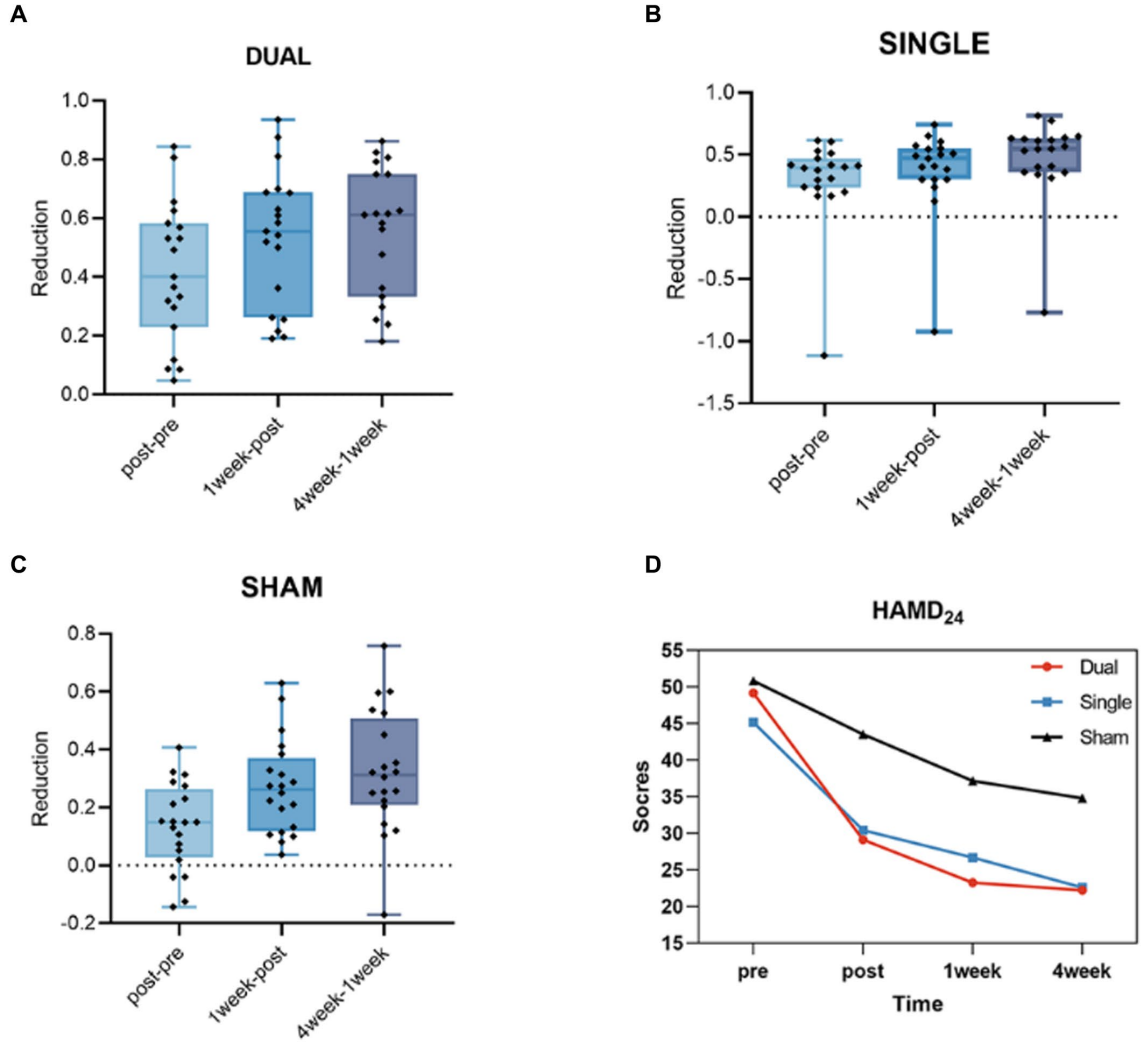
**(A)** The HAMD24 reduction rate from post- to pre-treatment, 1 week after treatment to post-treatment and 4 weeks after treatment to 1 week after treatment in the dual group. **(B)** The HAMD24 reduction rate from post- to pre-treatment, 1 week after treatment to post-treatment and 4 weeks after treatment to 1 week after treatment in the single group. **(C)** The HAMD24 reduction rate from post- to pre-treatment, 1 week after treatment to post-treatment and 4 weeks after treatment to 1 week after treatment in the sham group. **(D)** The average HAMD24 scores of pre-treatment, post- treatment, 1 week and 4 weeks after treatment in dual, single and sham groups.

At baseline, the HAMD24 scores in the dual, single, and sham groups were not significantly different (*p* > 0.05). After 5 days of aTMS treatment, the HAMD24 scores were reduced in these three groups. Additionally, this decrease persisted at 1 week and even 4 weeks after aTMS treatment. After the treatment, the reduction rates in HAMD24 scores in dual, single and sham group were 41.67% ± 0.239, 29.70% ± 0.368 and 13.43% ± 0.151, respectively. At 1 week after treatment, the rate were decreased by 53.24% ± 0.231, 37.72% ± 0.350 and 27.00% ± 0.162, respectively. At 4 weeks after treatment, the rates were decreased by 55.47% ± 0.219, 47.17% ± 0.333 and 32.49% ± 0.211, respectively. These reduction rates of HAMD24 scores in three groups were significant different (*p* < 0.05). Additionally, the reduction rate was faster and greater in the dual group than in the other two groups (Table 1, Figure 1).

### 3.2 ALFF and fALFF analysis

**Figure 2:**
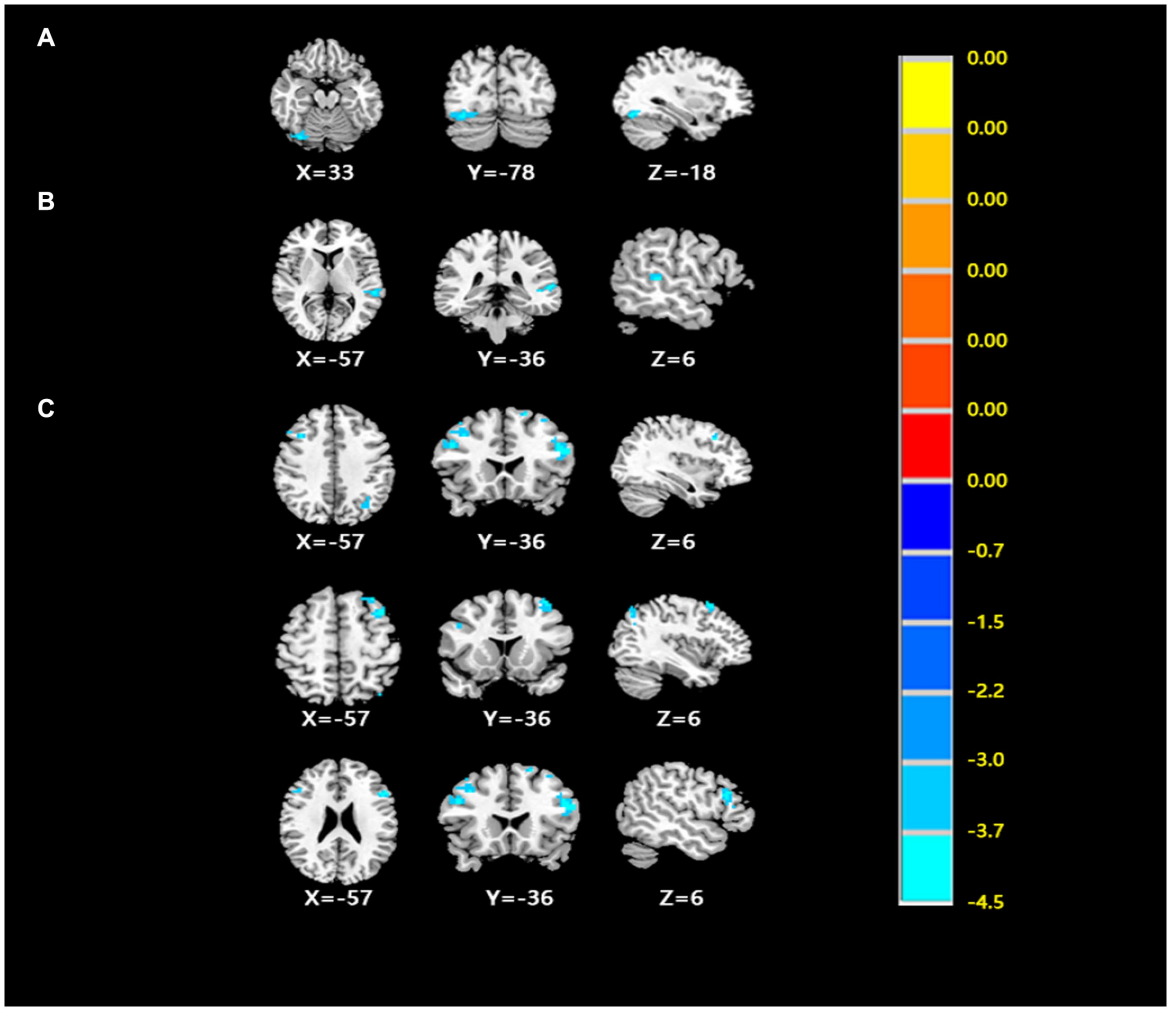
**(A)** The differences of ALFF from post- to pre- treatment in the dual group. **(B)** The differences of fALFF from post- to pre- treatment in the dual group. **(C)** The differences of FC from post- to pre- treatment in the dual group. (Cluster *p* < 0.05, GRF corrected) (Cluster *p* < 0.05, GRF corrected).

**Figure 3:**
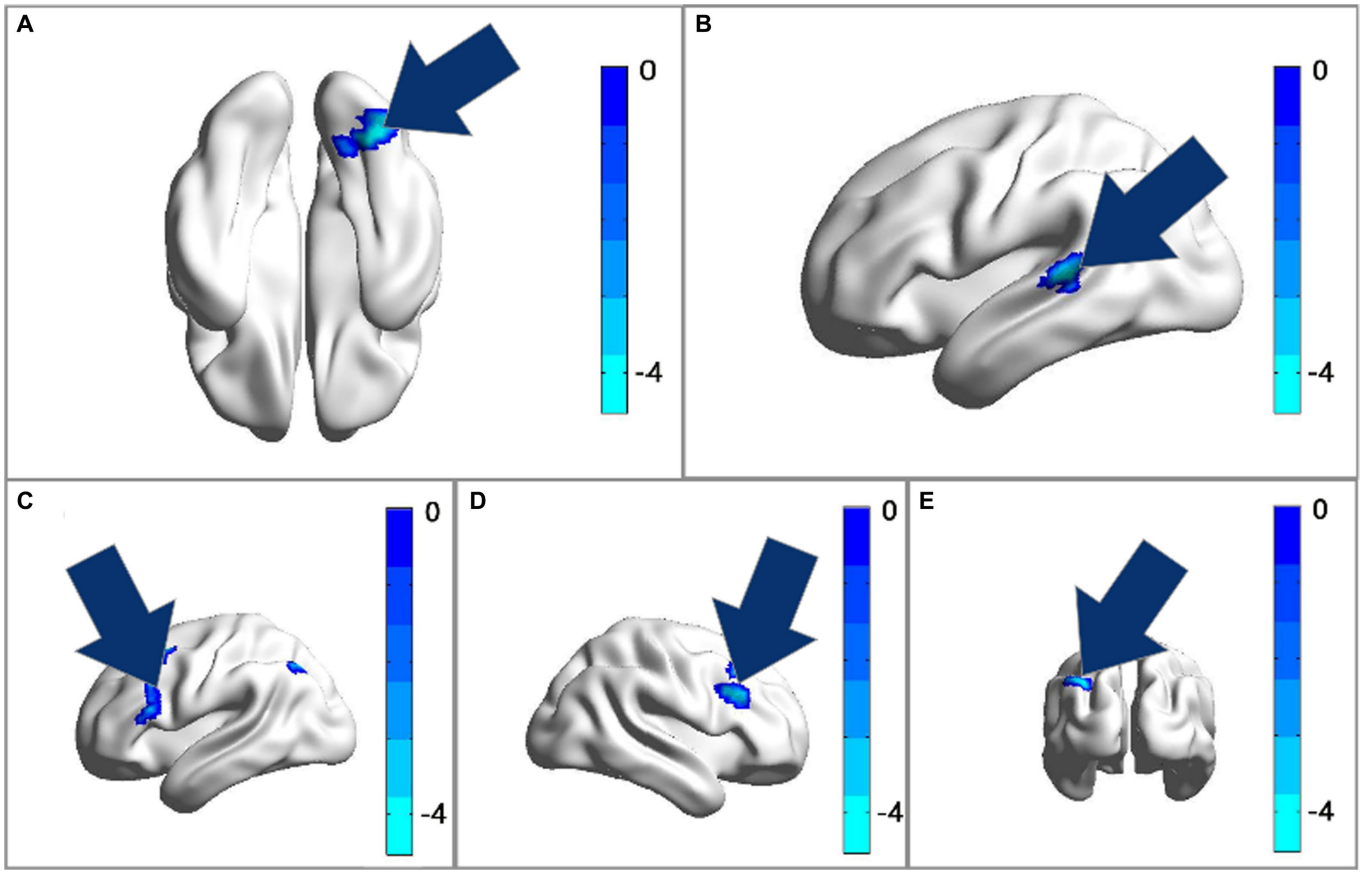
**(A)** The ALFF analysis revealed significant reductions in the right fusiform gyrus after treatment. **(B)** The fALFF analysis revealed significant reductions in the left superior temporal gyrus after treatment with GRF correction. **(C–E)** The FC of ROI1 (33, −78, −18) with the bilateral middle frontal gyrus and triangular part of inferior frontal gyrus had decreased after treatment. Cool colors represent decreased value, while warm colors represent increased value. These differences were obtained by paired t-test in the dual group.

After comparing the post- and pre-TMS images by the paired *t*-test, the ALFF value in the right fusiform gyrus (FG) (peak MNI coordinate: 33, −78, −18) of the dual group was found to decrease after aTMS. Additionally, the left superior temporal gyrus (STG) (peak MNI coordinate: −57, −36, 6) in the dual group had a lower fALFF value after aTMS. In both the single and sham groups, there were no significant changes in ALFF and fALFF values in the whole brain before and after aTMS (Table 2, Figures 2A,B, 3A,B).

**Table 2:** Brain regions alterations of ALFF, fALFF, and FC in TRD patients after arTMS in dual group.*

Brain regions	Left/Right	Peak MNI coordinate			Clusters (voxel)	Peak T value
		x	y	z		
*Post* < *hoc*						
ALFF						
Fusiform Gyrus ^a^	R	33	−78	−18	41	−5.5831
fALFF						
Superior Temporal Gyrus ^b^	L	−57	−36	6	42	−6.4519
FC of seed1: right fusiform gyrus (Peak MNI: 33–78 -18)						
Middle Frontal Gyrus ^c^	R	36	21	39	71	−4.7969
Middle frontal gyrus^c^	L	−39	15	57	59	−6.3005
Inferior frontal gyrus, triangular part^c^	L	−51	21	24	52	−5.8810
FC of seed2: left superior temporal gyrus (Peak MNI: −57 -36 6)						
NONE						

ALFF, Low-frequency Fluctuation; fALFF, Fractional Amplitude of Low-frequency Fluctuations. FC, Functional Connectivity. MNI, Montreal Neurological Institute. HAMD24, Hamilton Depression Scale-24. The peak MNI coordinate represent the peak points with most significant differences in the brain areas.

Gaussian Random Field (GRF) correction: voxel *p* value = 0.002, cluster *p* value = 0.1, two tail (Z > 2.3, cluster *p* = 0.05, one tail).

*There was no significant result in both single and sham group after the GRF correction.

^a^The result of ALFF analysis.

^b^The result of fALFF analysis.

^c^The result of ROI analysis with the seed of right Fusiform Gyrus.

### 3.3 ROI analysis

The peak MNI coordinates for the ALFF and fALFF values (33, −78, −18 and − 57, −36, 6) were selected as ROIs to explore the FC between these two ROIs with whole brain. We found that in the dual group, after 5 days of aTMS treatment, the FC between ROI1 (33, −78, −18) and the right middle frontal gyrus (MFG), left MFG, left triangular part of inferior frontal gyrus (IFG) decreased. There was no significant difference in the FC between ROI2 (−57, −36, 6) and the other brain regions before and after treatment (Table 2, Figures 2C, 3C–E).

### 3.4 Correlation analysis

**Figure 4:**
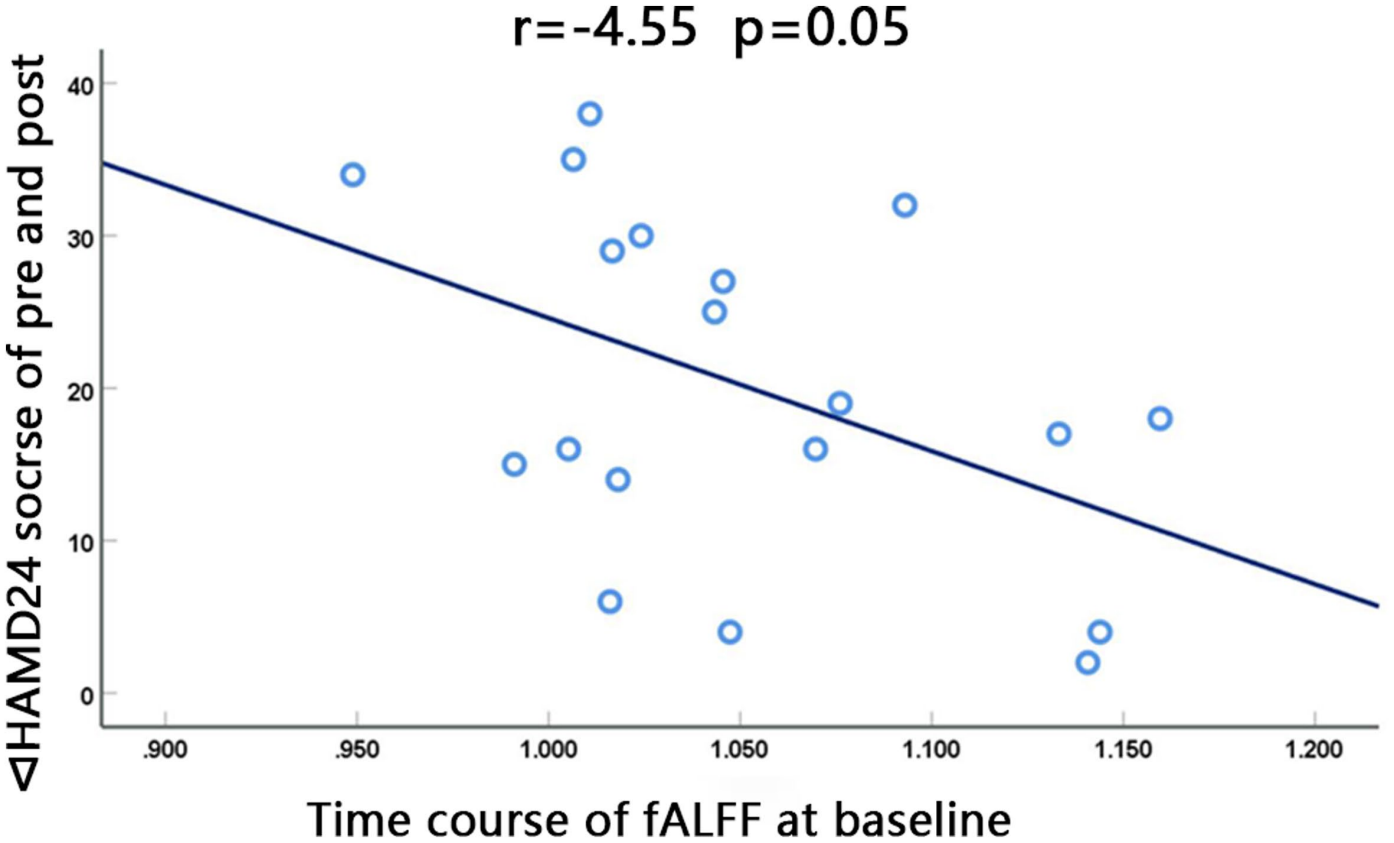
The fALFF value at baseline of the left superior gyrus in the dual group was negatively correlated with the difference between HAMD_24_ scores before and after treatment (∆HAMD_24_ score). (*r* = −0.455, *p* = 0.050, Pearson correlation).

The fALFF value of the left STG at baseline in the dual group was negatively correlated with the difference between HAMD24 scores before and after treatment (∆HAMD24 score) (*r* = −0.455, *p* = 0.050, Pearson correlation), and the ALFF value of the right FG was not significantly correlated with ∆HAMD24 score (Figure 4).

## 4 Discussion

The current study investigated the features of spontaneous brain activity in TRD pre- and post-aTMS treatment at different targets using rs-fMRI. Specifically, we researched the efficacy and safety of aTMS for TRD by performing bilateral, unilateral, or sham stimulation at left DLPFC and right OFC and then analyzed the ALFF, fALFF and FC values of different brain regions in those three groups before and after aTMS treatment.

The DLPFC is a key region of the executive control network (ECN) that is associated with the regulation of attention, decision-making, working memory, and cognitive control. Therefore, researchers believe that applying high-frequency stimulation to the left DLPFC could help reduce depressive symptoms and further improve the mood of patients with depression (39). Additionally, the OFC is also associated with emotions since it is reacting to reward values (40–42). A series of studies found that the OFC is a vital brain area for reward and is activated by unpleasant aversive stimuli. After the aTMS at these two targets, the depressed symptoms of TRD patients get significantly improved. These regions thus could be the strong candidate targets for stimulation to treat depression, particularly for the patients with TRD (43–46). Specifically, we found that both dual and single target stimulation reduced HAMD24 scores in the short term, indicating that both these two schemes of aTMS treatment can improve the symptoms of patients with TRD. Because the TMS could produce a strong magnetic field with repeated pulses that passes through the scalp and skull beneath the coil to enhance or weaken activity in corresponding brain area by different models of stimulation. According to the differences of frequencies, TMS stimulations are divided into low-frequency stimulation (≤ 1 Hz) and high-frequency stimulation (> 5 Hz) (47). The former and continuous theta burst stimulation (cTBS) or the latter and intermittent theta burst stimulation (iTBS) respectively execute inhibitory or excitatory effects on the brain cortex. An increasing number of related studies have also proven the effectiveness of TMS for depression. Such as a meta-analysis (48) that included 15 published articles on the use of repetitive transcranial magnetic stimulation (rTMS) to treat depression from 2001 to 2010 concluded that compared with sham stimulation, real rTMS targeting the left and right DLPFC with high- and low-frequency stimulation was effective in the treatment of depression. Besides, a recent study showed that the response rates to rTMS of patients with major depressive disorder (MDD) were 40 to 50%, and the remission rates were 25 to 30% (49). In our study, the dual group showed a faster and greater decrease than the single and sham group. In addition, after dual target stimulation, at 1 week after the treatment and at 4 weeks after the treatment, the reduction rates in HAMD24 scores were also increasingly greater in the dual group than that in the other two groups. Importantly, both dual and single target stimulation showed good safety and tolerability. In this study, none of the subjects experienced adverse reactions, such as severe headache, seizures, or hearing loss, during treatment.

Additionally, we used the ALFF to observe the fluctuation of the average amplitude of voxels in the frequency range of 0.01–0.08 Hz; this value directly indicates changes in the amplitude of the BOLD signal and reflects the spontaneous activity of brain regions (28). In this study, we found that after dual target aTMS, the ALFF value of the right FG was significantly lower than that before treatment. This finding is consistent with the results of a previous rs-fMRI study that revealed patients with depression tend to be with higher ALFF values in the right FG (50). Numerous rs-fMRI studies have found that changes in the FG of patients with depression suggested that the neurological activity of this brain region is altered, which may be the basis of depression (51–53). As well known, the FG is a part of the visual recognition network and temporal cortex, which is at the same time responsible for facial recognition and the deep processing of visual information as well as negative cognition and emotion. So, it may be the area to display the earliest signs of abnormal emotional processing in patients with depression (54–56). Besides, abnormal spontaneous brain activity of the FG may indicate impaired understanding and memory of language as well as recognition of facial features in MDD patients, which may lead to negative cognition and affect in both learning and life (57).

We also found that after aTMS treatment the FC of the right FG was decreased. Specifically, the FC between the right FG and bialetral MFG and left triangular part of IFG was decreased after treatment. Shan et al. also found that the FC of the right FG was abnormal in patients with depression, which may produce mood disorders (58, 59). And this kind of abnormal FC of right FG mainly focused on the frontal lobe which could divide into the supra, middle, and lower folds (60). Because it is one of the areas involved in the higher functional activities of the human brain, influencing social behavior, planning, language formation, working memory, language search, extraction, naming and other functional activities, it is closely related to many mental diseases. In addition, a study by Liu et al. also suggested that spontaneous brain activity in the right MFG of patients with depression is significantly correlated with depressive symptoms (61). One of our previous studies also indicated that the changed of the FC between right praecuneus and MFG was related to improvement of depressive symptoms after cognitive-behavioral therapy combined with drugs (62). The MFG is a core area of the DLPFC, which plays a key role in emotional supervision and cognitive processing (63). Hyperactivities in this area was observed in depressive patients compared to controls (64). As a part of DLPFC, the left IFG is extensively involved in language processing, working memory and cognitive control (65–68). When the processing of negative emotions increases, it can specifically inhibit the overworked limbic system by connecting with the orbitofrontal cortex, so that negative emotion processing is reduced (69). Conversely, when the processing of negative emotions is reduced, the functional connectivity between these two brains decreases.

The fALFF, obtained by dividing the energy of the low-frequency signal by the energy of the entire frequency band, is a common indicator of resting-state fMRI and can reduce the influence of noise in the data (70). Hence, in this study, we also used fALFF to observe the effective reduction of the intensity of spontaneous neuronal activity in brain regions. Related studies found that the fALFF of the left STG in patients with depression was significantly increased (71, 72). After aTMS, we found that the fALFF of the left STG, decreased compared to that at baseline in the dual group, which is consistent with the results of other studies (73, 74). Moreover, fALFF value of the left STG at baseline in the dual group was negatively correlated with the ∆HAMD24 score before and after treatment. Some fMRI studies also have showed alterations in the STG in patients with depression (73, 74). The STG is a critical part of temporal lobe which is mainly responsible for not only processing auditory information but also advanced neural activities such as social cognition (75), Some studies have found that the STG and its adjacent cerebral cortex played an important role in processing information related to individual communication (such as eye gaze direction, facial expression, and lip movements). Thus, it may be mainly responsible for the dynamic processing of facial features, which is more important during individual communication (76).

The STG and FG, as part of the temporal-occipital junction, were reported to be more sensitive to negative emotional information (77). And after this kind of protocol of aTMS treatment, it took a short period to improve the abnormal ALFF values and FC in FG as well as abnormal fALFF valued in STG and then to reduce this sensitivity, thereby helping to improve the negative mood of patients with TRD.

## 5 Conclusion

This study has demonstrated that the efficacy of the dual target treatment was better than that of the single-target and sham treatments. In addition, we also demonstrated that the functional disorder of the right FG and left STG, which could be significantly improved after aTMS treatment, may be the pathological bases of emotional and cognitive disorder in depression. And these areas may indicate the potential marker of efficacy of dual target aTMS treatment. Particularly, higher baseline fALFF values in the left STG may suggest better response for dual target aTMS treatment. These findings may help improve the understanding of neurobiological mechanism of TRD.

## 6 Limitations and future directions

This study has the following limitations: firstly, the sample size is small which should be further expanded in future studies. Secondly, a precise navigation system was not used. This may result in some errors due to insufficient anatomical data support and failure to consider individual differences. Thirdly, it wasn’t made an assessment of what might be a protective factor through psychotherapy and counseling intervention during the survey. In future research, we will further improve these shortcomings.

## Data availability statement

The raw data supporting the conclusions of this article will be made available by the authors, without undue reservation.

## Ethics statement

The studies involving humans were approved by Ethics Committee of the Second People’s Hospital of Guizhou. The studies were conducted in accordance with the local legislation and institutional requirements. The participants provided their written informed consent to participate in this study.

## Author contributions

JL: Formal analysis, Methodology, Visualization, Writing – original draft. YS: Funding acquisition, Methodology, Project administration, Resources, Supervision, Writing – review & editing. GW: Methodology, Project administration, Resources, Supervision, Writing – review & editing. LH: Investigation, Methodology, Writing – review & editing. HC: Investigation, Methodology, Writing – review & editing.

## Footnotes

^1^ http://www.restfmri.net

^2^ http://www.nitrc.org/projects/bnv/
